# Analysis of the genetic association between *IL27* variants and coronary artery disease in a Chinese Han population

**DOI:** 10.1038/srep25782

**Published:** 2016-05-12

**Authors:** Qian Fan, Shaofang Nie, Sihui Li, Yuhua Liao, Hongsong Zhang, Lingfeng Zha, Fan Wang, Tingting Tang, Ni Xia, Chengqi Xu, Pengyun Wang, Tian Xie, Jiangjiao Xie, Qiulun Lu, Qingxian Li, Jin Qian, Bin Li, Gang Wu, Yanxia Wu, Yan Yang, Qing K. Wang, Xin Tu, Xiang Cheng

**Affiliations:** 1 Laboratory of Cardiovascular Immunology, Institute of Cardiology, Union Hospital, Tongji Medical College, Huazhong University of Science and Technology, Wuhan, China; 2 Department of Biostatistics and Epidemiology, University of Pennsylvania, Philadelphia, USA; 3 Key Laboratory of Molecular Biophysics of Ministry of Education, College of Life Science and Technology, Center for Human Genome Research, Cardio-X Institute, Huazhong University of Science and Technology, Wuhan, China; 4 Section of Molecule Medicine, Department of Medicine, University of Oklahoma Health Sciences Center, Oklahoma City, USA; 5 Department of Cardiology, Jining Medical College Affiliated Hospital, Jining, China; 6 Department of Cardiology, Suizhou Central Hospital, Suizhou, China; 7 Department of Cardiology, Xiangyang Central Hospital, Xiangyang, China; 8 Renmin Hospital of Wuhan University, Wuhan, China; 9 Wuhan No. 1 Hospital, Wuhan, China

## Abstract

Interleukin-27 (IL-27) is an important cytokine in inflammatory diseases, including coronary artery disease (CAD). To explore the precise role of IL-27 in CAD, we investigated the genetic association between *IL27* and CAD in the GeneID Chinese Han population. A two-stage case control association analysis was performed for 3075 CAD cases and 2802 controls. Logistic regression analysis was used to adjust the traditional risk factors for CAD. Results showed that a promoter variant, rs153109, tended to be marginally associated with CAD in the discovery population (*P*_adj_ = 0.028, OR = 1.27, 95%CI: 1.03–1.58). However, this association was not replicated in the validation stage (*P*_adj_ = 0.559, OR = 1.04, 95%CI: 0.90–1.21). In addition, when we classified the combined population into two subgroups according to the age at disease onset or disease state, we again obtained no significant associations. Finally, we estimated the severity of coronary stenosis using the Gensini Scoring system and determined that the rs153109 genotypes were still not associated with the Gensini scores of the CAD patients. In conclusion, our study failed to find an association between common variants in the functional region of *IL27* and CAD in a Chinese Han population, which indicated that IL-27 might only be an inflammatory marker during the development of CAD.

Coronary artery disease (CAD), the leading cause of death and infirmity worldwide, is a complex disease caused by multiple genetic and environmental factors, as well as the interactions between them. Although genome-wide association analysis studies have identified more than 50 risk loci for CAD in recent years^1^,^2^,^3^, the heritability of CAD is still incompletely understood^4^,^5^. Therefore, more studies are urgently needed to identify the genetic factors to fully explain the molecular genetic mechanisms of CAD and provide valuable suggestions for the prevention and treatment of CAD.

Atherosclerosis is the basis for CAD pathogenesis. Inflammation plays an important role and is involved in the initiation and progression of atherosclerosis^6^. Recent studies show that genetic variations in inflammatory cytokines are involved in the process, including *IL6*, *IL16*, *IL17A*, *CRP*, and *IL33*^2^,^7^,^8^,^9^,^10^. IL-27 is a new member of the IL-6/IL-12 family and comprises an Epstein-Barr virus-induced molecule 3 (EBI3) subunit joined with the p28 subunit^11^. IL-27 plays an important role in the innate and adaptive immune systems. Variants in the *IL27* gene contribute to the risk of multiple inflammatory immune diseases, such as inflammatory bowel disease, rheumatoid arthritis, asthma, and chronic obstructive pulmonary disease^12^,^13^,^14^,^15^,^16^. However, the role of the IL-27 in atherosclerosis is uncertain. In 2011, Jafarzadeh *et al.* reported that the serum level of IL-27 was elevated in patients with ischemic heart diseases^17^. Later, Jin *et al.* found that elevated level of IL-27 in the circulation of CAD patients was significantly associated with the level of ox-LDL and the severity of coronary artery stenosis, as estimated by the Gensini Scoring system^18^. This evidence indicates that IL-27 might play a causal role in the development of CAD. However, in contrast, some studies found that IL-27 and its receptor could inhibit the activity of macrophages and may retard the process of atherosclerosis^19^,^20^. These controversial findings regarding the relationship between IL-27 and CAD prompted us to investigate the genetic role of *IL27* in CAD, which might provide scientific evidence to explain the exact function of IL-27 in the development of CAD.

Here, we performed a two-stage association analysis to determine the relationship between common *IL27* variants and CAD in a Chinese Han population and further classified the combined populations according to the age at disease onset and the disease state. Additionally, the association between rs153109 in the *IL27* gene and the severity of CAD was investigated.

## Results

### Characteristics of the population

Table 1 illustrates the detailed clinical features of the two populations enrolled in this study. As expected, the age, BMI, TG, Tch and LDL-c levels were significantly higher in the CAD patients than in the controls. The male percent and the prevalence of smoking, hypertension and diabetes mellitus were also significantly increased in the CAD patients compared with the controls.

With an effect size of 1.2 and a minor allele frequency based on the Hap Map CHB and JPT datasets, the statistical power for all of the studied variants was greater than 50% in the discovery populations and greater than 95% in the validation populations. The combined sample size also provided a statistical power of greater than 100% for rs153109.

### Association analysis of four tag SNPs in the GeneID Chinese Han population

All selected variants passed the Hardy-Weinberg equilibrium test, with *P* values greater than 0.001 in the control subjects. In the discovery stage, the allelic frequency of rs153109 tended to be marginally associated with CAD after adjusting for age, sex, smoking, BMI, hypertension, diabetes mellitus, and the lipid concentrations (rs153109^C^, *P*_adj_ = 0.028, OR = 1.27, 95%CI: 1.03–1.58). The other three variants were not associated with CAD (rs181206^G^, *P*_adj_ = 0.719, OR = 0.93, 95%CI: 0.64–1.36; rs17855750^C^, *P*_adj_ = 0.229, OR = 1.22, 95%CI: 0.88–1.69; rs34833^T^, *P*_adj_ = 0.247, OR = 1.17, 95%CI: 0.90–1.54) (Table 2). Using the genotypic association analysis, there was no significant association between the three SNPs (rs181206, rs17855750 and rs38433) and CAD (*P*_adj_ > 0.05) (Table 3). Thus, no further analysis was performed on these variants. In the validation cohort, the association between rs153109 and CAD was not further verified after adjusting for covariates (rs153109, *P*_adj_ = 0.559, OR = 1.04, 95%CI: 0.90–1.21). After combining the two cohorts, we performed a combined association analysis (3075 CAD cases *vs* 2802 controls) between rs153109 and CAD, which also showed that there was no significant association (rs153109, *P*_adj_ = 0.164, OR = 1.08, 95%CI: 0.97–1.21). Moreover, no significant genotypic association was identified in different cohorts (Tables 2 and 3).

### Stratified analysis for the association between rs153109 and CAD by the age at disease onset and disease state in the GeneID-combined Population

An additional statistical analysis was performed by dividing the total CAD patients into two subgroups by the age at disease onset: one is the early-onset CAD subgroup with 847 individuals, and the other is the late-onset CAD subgroup with 2157 subjects. The early-onset CAD group was defined as having an age of CAD onset of less than 55 years for male patients and less than 65 years for female patients^21^. Using the allelic and genotypic association analyses, there were no significant associations between rs153109 and CAD in the early-onset and late-onset subgroup populations after adjusting for the risk factors (*P*_adj_ > 0.05 for both the allelic and genotypic associations) (Tables 4 and 5). The Breslow–Day test for heterogeneity was not significant (*P* = 0.844), suggesting the homogeneity of different disease onset age groups.

In addition, we divided the CAD population into two subgroups by the disease state: one is the anatomical-CAD group, consisting of approximately 1205 CAD subjects with severe coronary stenosis, and the other is the clinical-CAD group, which includes 1799 CAD subjects with myocardial infarction or revascularization^2^. The results showed that rs153109 was not significantly associated with CAD in both subgroups after adjusting for the risk factors (*P*_adj_ > 0.05 for the allelic and genotypic associations) (Tables 4 and 5). The Breslow–Day test for heterogeneity was not significant (*P* = 0.335), suggesting the homogeneity of different disease state groups.

### Analysis of the association between rs153109 and the Gensini scores in the GeneID-CAD population

**Figure 1:**
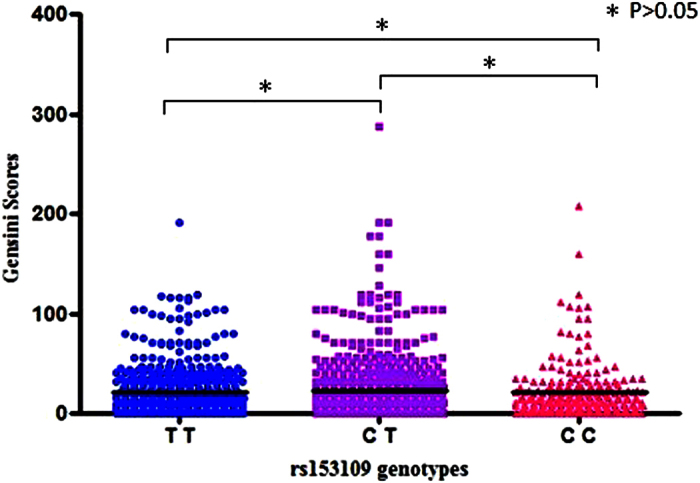
Association analysis between the Gensini scores and rs153109 genotypes. The comparison between the Gensini scores and the rs153109 genotypes via the Mann-Whitney *U*-test was shown. A solid line indicates the median value of the Gensini score.

We further investigated the possible association of rs153109 in *IL27* with the severity of CAD, as estimated by the Gensini Scoring system, in 1488 CAD cases who had received a coronary angiography examination. After log_e_-transformation, we performed a quantitative trait association analysis and a quartile case control association analysis between the genotypes of rs153109 and the Gensini scores. Unfortunately, rs153109 was not statistically correlated with the severity of atherosclerosis, as estimated by the Gensini scores (Table 6 and Fig. 1).

## Discussion

In this study, we aimed to evaluate the contribution of polymorphisms in the *IL27* gene to CAD susceptibility in Chinese Han subjects by employing a two-stage case control association analysis. Our study demonstrated that none of the variants from the functional region were different between the CAD cases and control subjects. IL-27, a heterologous dimer composed of EBI3 and p28, was shown to promote CD4+ T cell proliferation^22^. IL-27R is the orphan receptor of IL-27 and is expressed in a variety of inflammatory immune cells such as monocytes, macrophages, dendritic cells, mast cells, NK cells, endothelial cells, and T/B lymphocytes^23^,^24^,^25^,^26^. It was confirmed that IL-27 could regulate the proliferation, differentiation and maturation of multiple immune cells and was involved in inflammatory immune diseases^27^,^28^,^29^,^30^,^31^. Epidemiological studies have suggested that the serum IL-27 levels were elevated in patients with multiple sclerosis, psoriasis, Behcet’s disease, and visceral leishmaniasis but were attenuated in patients with rheumatoid arthritis and systemic lupus erythematosus disease^32^,^33^,^34^,^35^,^36^,^37^. Some clinical experiments revealed that IL-27 could prolong survival of patients with glomerulonephritis and improve the joint pathology of patients with arthritis but aggravated airway inflammation in allergic asthma patients^38^,^39^,^40^. Additionally, new studies also found that genetic polymorphisms in *IL27* were significantly associated with multiple inflammatory immune diseases. These results suggested that IL-27 might play important roles in the pathogenesis of inflammatory autoimmune diseases, but the molecular mechanisms are probably quite different.

From 2011 to 2013, several researchers were involved in uncovering the relationship between IL-27 and CAD and reported that IL-27 might play a dual role in promoting and inhibiting different stages of the development of CAD. The contradictory findings for the role of IL-27 in the development of CAD in previous studies might be explained by an unknown biological interaction between IL-27 and other known or unknown CAD risk factors. In our genetic research, we demonstrated that the allelic and genotypic frequencies were not associated with the susceptibility to CAD in a Chinese Han population, even though the rs153109 variant tended to be marginally associated with CAD in the discovery stage. In addition, a subsequent analysis also found that the association between rs153109 and CAD was not significant in the different stratifications and stenosis severities. Recently, another study indicated that variants in *IL27* did not differ between T1D patients and the controls in a Brazilian population, even when these variants were analyzed together with the major HLA-DRB1 risk alleles^41^. Another study also found that a genetic variant at the *IL27* locus was associated with T1D, which had a strong cis-eQTL effect on CCDC101 instead of the *IL27* gene^42^. Interestingly, CAD and T1D are both metabolic diseases, and an imbalance in Th1/Th2 cell function and a disrupted oxidative stress response play important roles in the pathogenesis of these diseases. Based on the above evidence, we might conclude that the inflammatory cytokine IL-27 was only an inflammatory marker during the development of CAD.

There were some disadvantages in our study. First, the relatively small number of subjects in this study might lead to false negative association results. Second, the studied subjects were enrolled from different centers in China and might have different environmental and genetic risks. Third, although we selected tagged SNPs in the functional region of *IL27*, more variants in the non-coding regions surrounding the gene or between exons might be missed. Finally, some control subjects might develop cardiovascular diseases in the future.

In conclusion, although *IL27* is an attractive candidate that may contribute to inflammatory immune diseases, our study failed to find an association between common variants in the functional region of *IL27* and CAD in a Chinese Han population. However, this finding needs to be confirmed in a larger sample and in different ethnic groups.

## Methods

### Study Population

A two-stage case control genetic association study was performed with a total of 5877 subjects (3075 CAD cases *vs* 2802 controls). All subjects were enrolled from the GeneID Chinese Han population, which is a large ongoing database that aims to study the genetic basis of cardiovascular diseases. In the first stage, 2322 subjects (1245 CAD cases and 1077 controls) from the GeneID-Central-China population were enrolled from Hubei province and served as the discovery cohort. In the second stage, 3555 subjects (1830 CAD cases and 1725 controls) from the GeneID-Northern-China population were enrolled from Shandong and Liaoning provinces and served as the validation cohort. The criterion for the enrollment as a CAD case was defined as a stenosis diameter of 70% in any of the main coronary arteries (left main, left anterior descending, left circumflex artery or right coronary artery) by coronary angiography, a coronary artery bypass graft, percutaneous coronary intervention, and/or myocardial infarction. Subjects who had experienced myocardial spasms or had a myocardial bridge, as identified by angiography, or had congenital heart disease, cardiomyopathy, heart valve disease, renal or hepatic disease, and autoimmune diseases were excluded from the study. The control subjects were selected from individuals whose major coronary artery displayed no more than 30% stenosis as confirmed by angiography and who had no history of CAD. The disease states of hypertension and diabetes mellitus were evaluated according to published guidelines^43^,^44^. The fasting total cholesterol (Tch), triglyceride (TG) and LDL cholesterol (LDL-c) concentrations were measured using standard methods^2^,^45^. Direct interviews and medical record reviews were performed to collect the subjects’ clinical characteristics, such as age, gender, body mass index (BMI), and smoking history. This study was approved by the Medical Ethical Committee of Huazhong University of Science and Technology and complied with the ethical principles set forth by the Declaration of Helsinki. All participants provided the informed consent.

### Gensini scores

The severity of coronary artery stenosis was estimated using the Gensini Scoring system^46^,^47^. For each segment, the score was rated as 1, 2, 4, 8, 16 and 32 for coronary stenosis of 0–25%, 26–50%, 51–75%, 76–90%, 91–99% and 100%, respectively. After multiplying the factor assigned to each segment with the vessel size and importance (ranging from 0.05 to 5.0), the Gensini index for 1488 cases undergoing conventional angiography was calculated by adding the total weights for each segment.

### Genetic Analysis

**Figure 2:**
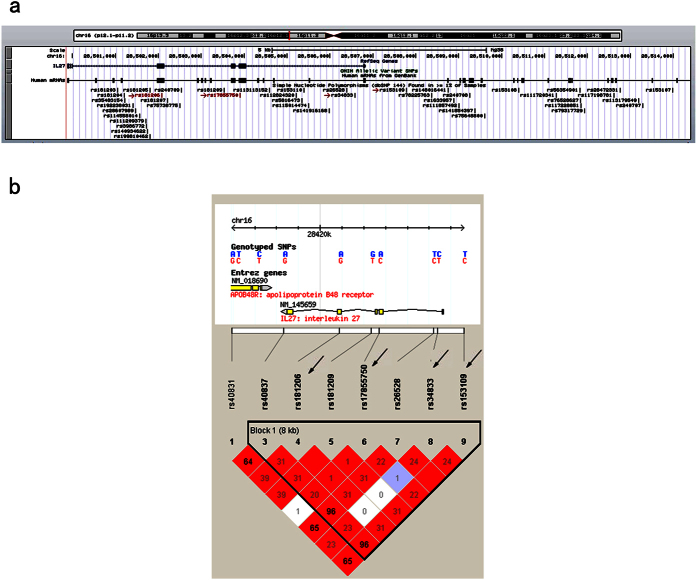
Regional plots based on the UCSC data sets and LD block based on the HapMap CHB and JPT data sets of *IL27*. (**a**) The association result was shown against the map position for each SNP within the region of 15420 bp. The marker SNPs of the associations are shown in arrow. Red SNPs represent non-synonymous. The annotated gene within the critical region of the association is shown on the top. (**b**) The LD block of *IL27* was shown. The arrows indicate the selected tag SNPs. Each diamond represents the LD degree between the SNPs. The color indicates the *D*′ (a redder color represents a higher *D*′), and the numbers within the diamonds are the *r*^2^ values.

The DNA samples were collected from the peripheral blood. The map position within the region of 15420bp was shown in the regional plot (Fig. 2). The tag SNPs were selected according to the following principles: (1) construction of a linkage disequilibrium (LD) map with the single nucleotide polymorphisms (SNPs) of *IL27* using Haploview (v.4.2) and the HapMap CHB and JPT datasets (v.3, release 2), with thresholds of *r*^2^ > 0.8 to reduce redundancy; (2) variants with a minor allele frequency of more than 0.05; (3) functional variants reported by previous studies; (4) variants located in the promoter region or exon region; (5) and the LD block was analyzed using the four-gamete rule. Finally, four tag SNPs (rs181206, rs17855750, rs34833 and rs153109) that completely covered the *IL27* gene were selected. rs181206 and rs17855750 are located in the exon region, which might affect the function of the *IL27* protein. rs34833 and rs153109 are located in the predicted promoter region, which might regulate the expression of *IL27* (Fig. 2).

All of the subjects in this study were genotyped using a Rotor-Gene 6000 High-Resolution Melt (HRM) system (Corbett Life Science, Concorde, NSW, Australia). Genotyping was performed by PCR in a total reaction volume of 25 μl PCR, containing 0.7 μl of LC Green dye, 10 pmol of each primer, 30 ng of genomic DNA, 2.5 μl of 10× PCR buffer with 1.5 mmol/L MgCl_2_, 5 mmol deoxynucleotide triphosphates, and 1 unit of Taq polymerase. The genotyping results were verified by direct DNA sequencing analysis with 48 cases and 48 controls randomly selected from the studied subjects. The success rates for the different SNPs ranged from 92.9 to 100%.

### Statistical Analysis

The Hardy-Weinberg disequilibrium test was performed on the controls (plink). The allelic and genotypic association analyses were performed using Pearson’s 2 × 2 and 2 × 3 contingency table chi-square tests; the odds ratio (OR) and 95% confidence interval (CI) were also calculated (SPSS, v.17.0). Age, gender, BMI, hypertension, diabetes mellitus, smoking history, Tch, TG, and LDL-c were studied and analyzed as covariates using multiple logistic regression analysis^48^. Breslow-Day tests were used to assess the heterogeneity from different subgroups using SPSS (version 17.0). The statistical power analysis was performed with a free program that calculates the sample size and power (PS v.3.0.12)^2^. After log_e_-transformation, the Gensini scores were analyzed using a linear regression analysis and a quartile case control association analysis.

## Additional Information

**How to cite this article**: Fan, Q. *et al.* Analysis of the genetic association between *IL27* variants and coronary artery disease in a Chinese Han population. *Sci. Rep.* **6**, 25782; doi: 10.1038/srep25782 (2016).

## Figures and Tables

**Table 1:** Clinical characteristics of the studied GeneID Chinese Han population.

Characteristics	GeneID-discovery		*p*	GeneID-validation		*p*	GeneID-combined		*p*
	CAD	Control		CAD	Control		CAD	Control	
Subject numbers	1245	1077	–	1830	1725	–	3075	2802	–
Age (years)	63.0 ± 11.2	42.4 ± 11.2	3.40 × 10^−307^	66.7 ± 12.4	59.9 ± 11.6	3.00 × 10^−62^	65.2 ± 12.1	53.1 ± 14.2	2.14 × 10^−239^
Gender (male %)	70.1	59.2	1.18 × 10^−7^	64.4	59.2	1.32 × 10^−3^	66.7	59.2	8.92 × 10^−9^
Smoking (%)	44.7	16.9	8.88 × 10^−47^	26.7	19.7	3.11 × 10^−4^	34.2	18.0	3.53 × 10^−33^
BMI (kg/m^2^)	24.2 ± 1.59	23.7 ± 1.33	2.18 × 10^−12^	24.4 ± 0.88	23.9 ± 1.64	6.66 × 10^−38^	24.3 ± 1.22	23.8 ± 1.53	2.22 × 10^−45^
Hypertension (%)	66.3	15.7	1.35 × 10^−133^	71.6	43.8	4.99 × 10^−37^	69.4	26.4	4.61 × 10^−180^
DM (%)	34.7	3.40	3.91 × 10^−78^	23.1	13.2	5.61 × 10^−8^	27.9	7.20	8.61 × 10^−66^
Tch (mmol/l)	5.45 ± 1.28	4.79 ± 0.72	9.44 × 10^−52^	4.44 ± 1.00	4.21 ± 0.70	2.14 × 10^−15^	4.85 ± 1.23	4.43 ± 0.76	8.25 × 10^−55^
TG (mmol/l)	1.69 ± 1.09	1.51 ± 1.20	1.28 × 10^−4^	1.61 ± 1.00	1.48 ± 0.58	1.59 × 10^−6^	1.65 ± 1.04	1.49 ± 0.87	9.26 × 10^−10^
LDL-c (mmol/l)	2.99 ± 1.03	2.75 ± 0.63	1.85 × 10^−12^	2.64 ± 0.81	2.42 ± 0.63	1.06 × 10^−19^	2.78 ± 0.92	2.55 ± 0.65	1.48 × 10^−30^

The data are presented as the mean ± standard deviation or a percentage; CAD, coronary artery disease; Tch, total cholesterol; TG, triglyceride; LDL-c, low-density lipoprotein cholesterol.

**Table 2:** Allelic association analysis between *IL27* and CAD in the GeneID Chinese Han population.

Population	SNP-allele	N		MAF		*P*_hwe_	*P*_obs_	*P*_adj_	OR (95%CI)
		Cases	Controls	Cases	Controls				
GeneID-discovery	rs181206^G^	1245	1077	0.082	0.086	0.254	0.605	0.719	0.93 (0.64–1.36)
	rs17855750^C^	1224	1068	0.108	0.109	0.205	0.899	0.229	1.22 (0.88–1.69)
	rs34833^T^	1183	975	0.194	0.180	0.914	0.244	0.247	1.17 (0.90–1.54)
	rs153109^C^	1174	982	0.412	0.385	0.589	0.078	0.028	1.27 (1.03–1.58)
GeneID-validation	rs153109^C^	1830	1725	0.397	0.375	0.072	0.065	0.559	1.04 (0.90–1.21)
GeneID-combined	rs153109^C^	3004	2707	0.403	0.379	0.072	0.010	0.164	1.08 (0.97–1.21)

SNP, single nucleotide polymorphism; CAD, coronary artery disease; MAF, minor allele frequency; *P*_hwe_, P value from the Hardy-Weinberg equilibrium tests; *P*_obs_, observed P value; *P*_adj_, P value adjusted by the covariates; OR, odds ratio after adjustment.

**Table 3:** Genotypic association of the tag SNPs in *IL27* with CAD in the GeneID Chinese Han population.

Population (n, case/control)	SNP-allele	Model	Cases	Controls	*P*_obs_	*P*_adj_	OR (95%CI)
GeneID-discovery (1245/1077)	rs181206^G^	ADD	7/191/1047	11/166/900	0.456	0.720	0.93 (0.64–1.36)
		DOM	198/1047	177/900	0.795	0.919	0.98 (0.66–1.47)
		REC	7/1238	11/1066	0.210	0.218	0.33 (0.06–1.94)
	rs17855750^C^	ADD	21/221/982	17/199/852	0.935	0.237	1.21 (0.88–1.67)
		DOM	242/982	216/852	0.831	0.224	1.25 (0.87–1.78)
		REC	21/1203	17/1051	0.817	0.730	1.22 (0.39–3.81)
	rs34833^T^	ADD	36/389/758	32/287/656	0.265	0.225	1.19 (0.90–1.59)
		DOM	425/758	319/656	0.142	0.165	1.25 (0.91–1.72)
		REC	36/1147	32/943	0.754	0.902	0.94 (0.35–2.51)
	rs153109^C^	ADD	180/607/387	141/474/367	0.103	0.024	1.29 (1.04–1.61)
		DOM	787/387	615/367	0.033	0.055	1.36 (1.00–1.87)
		REC	180/994	141/841	0.564	0.085	1.45 (0.95–2.20)
GeneID-validation (1830/1725)	rs153109^C^	ADD	275/902/653	225/845/655	0.152	0.554	1.05 (0.90–1.21)
		DOM	1177/653	1070/655	0.157	0.813	1.03 (0.83–1.26)
		REC	275/1555	225/1500	0.089	0.413	1.13 (0.85–1.50)
GeneID-combined (3075/2802)	rs153109^C^	ADD	455/1509/1040	366/1319/1022	0.030	0.157	1.09 (0.97–1.22)
		DOM	1964/1040	1685/1022	0.014	0.297	1.09 (0.93–1.28)
		REC	455/2549	366/2341	0.086	0.193	1.16 (0.93–1.44)

*P*_obs_, observed P value; *P*_adj_, P value adjusted by the covariates; OR, odds ratio after adjustment; ADD, additive mode, rs181206_GG/GA/AA; rs17855750_CC/CT/TT; rs34833_TT/TC/CC; rs153109_CC/CT/TT; DOM, dominant mode, rs181206_GG + GA/AA; rs17855750_CC + CT/TT; rs34833_TT + TC/CC; rs153109_CC + CT/TT; REC, recessive mode, rs181206_GG/GA + AA; rs17855750_CC/CT + TT; rs34833_TT/TC + CC; rs153109_CC/CT + TT.

**Table 4:** Allelic association analysis of rs153109 in the different subgroups of the GeneID-combined population.

Population	SNP-allele	N		MAF		*P*_hwe_	*P*_obs_	*P*_adj_	OR (95%CI)
		Cases	Controls	Cases	Controls				
CAD-early-onset	rs153109^C^	847	2707	0.400	0.379	0.072	0.116	0.389	1.06 (0.93–1.22)
CAD-late-onset	rs153109^C^	2157	2707	0.404	0.379	0.072	0.013	0.330	1.07 (0.93–1.24)
CAD-anatomical	rs153109^C^	1205	2707	0.393	0.379	0.072	0.235	0.622	1.04 (0.90–1.20)
CAD-clinical	rs153109^C^	1799	2707	0.409	0.379	0.072	0.004	0.160	1.09 (0.97–1.24)

CAD, coronary artery disease; MAF, minor allele frequency; *P*_hwe_, P value from the Hardy-Weinberg equilibrium tests; *P*_obs_, observed P value; *P*_adj_, P value adjusted by the covariates; OR, odds ratio after adjustment.

**Table 5:** Genotypic association analysis of rs153109 in the different subgroups of the GeneID-combined population.

Population (n, case/control)	SNP-allele	Model	Cases	Controls	*P*_obs_	*P*_adj_	OR (95%CI)
CAD-early-onset (847/2707)	rs153109^C^	ADD	131/416/300	366/1319/1022	0.258	0.380	1.07 (0.93–1.23)
		DOM	547/300	1685/1022	0.220	0.634	1.05 (0.86–1.28)
		REC	131/716	366/2341	0.154	0.298	1.15 (0.88–1.51)
CAD-late-onset (2157/2707)	rs153109^C^	ADD	324/1093/740	366/1319/1022	0.034	0.320	1.08 (0.93–1.25)
		DOM	1417/740	1685/1022	0.013	0.562	1.06 (0.87–1.30)
		REC	324/1833	366/2341	0.136	0.262	1.18 (0.89–1.56)
CAD-anatomical (1205/2707)	rs153109^C^	ADD	182/584/439	366/1319/1022	0.388	0.617	1.04 (0.90–1.20)
		DOM	766/439	1685/1022	0.430	0.710	0.96 (0.78–1.18)
		REC	182/1023	366/2341	0.188	0.142	1.23 (0.93–1.63)
CAD-clinical (1799/2707)	rs153109^C^	ADD	273/925/601	366/1319/1022	0.010	0.149	1.10 (0.97–1.25)
		DOM	1198/601	1685/1022	0.003	0.153	1.14 (0.95–1.37)
		REC	273/1526	366/2341	0.119	0.411	1.11 (0.87–1.41)

*P*_obs_, observed P value; *P*_adj_, P value adjusted by the covariates; OR, odds ratio after adjustment. ADD, additive model, rs153109_CC/CT/TT; DOM, dominant model, rs153109_CC + CT/TT; REC, recessive model, rs153109_CC/CT + TT.

**Table 6:** Genotypic association analysis of rs153109 and the LN-transformed Gensini scores in 1488 CAD patients.

SNP-allele	Quantitative trait association					Case control association				
	beta	SE	*r*^2^	*P*_obs_	*P*_adj_	RAF (n)		*P*_obs_	*P*_adj_	OR (95%CI)
						1^st^(245)	4^th^(394)			
rs153109^C^	0.002	0.014	0.008	0.587	0.277	0.391	0.386	0.854	0.646	0.94 (0.74–1.21)

The 1st and 4th quartiles of the LN [Gensini score] distribution were used to perform the case control association analysis. The 1^st^ quartile was defined as the quartile with the lowest Gensini scores, and the 4^th^ quartile was defined as the quartile with the highest Gensini scores. *P*_obs_, observed P value; *P*_adj_, *P* value adjusted by the covariates; OR, odds ratio after adjustment; the *P*_adj_ values and OR values were obtained using a multivariate logistic regression analysis.
